# 三嗪基多孔有机材料的合成及在固相微萃取应用中的研究进展

**DOI:** 10.3724/SP.J.1123.2020.07036

**Published:** 2021-02-08

**Authors:** Zhuo WANG, Wenjin WANG, Shuaihua ZHANG, Chun WANG, Zhi WANG

**Affiliations:** 河北农业大学理学院化学系, 河北 保定 071001; Department of Chemistry, College of Science, Hebei Agricultural University, Baoding 071001, China; 河北农业大学理学院化学系, 河北 保定 071001; Department of Chemistry, College of Science, Hebei Agricultural University, Baoding 071001, China; 河北农业大学理学院化学系, 河北 保定 071001; Department of Chemistry, College of Science, Hebei Agricultural University, Baoding 071001, China; 河北农业大学理学院化学系, 河北 保定 071001; Department of Chemistry, College of Science, Hebei Agricultural University, Baoding 071001, China; 河北农业大学理学院化学系, 河北 保定 071001; Department of Chemistry, College of Science, Hebei Agricultural University, Baoding 071001, China

**Keywords:** 固相微萃取, 合成, 三嗪基多孔有机材料, solid phase microextraction (SPME), construction, triazine-based porous organic polymers (TPOPs)

## Abstract

三嗪基多孔有机材料(TPOPs)具有较大的比表面积、可调的孔道结构、较高的热和化学稳定性、丰富的*π*键体系等诸多优点,目前被广泛应用于气体储存、催化、能源转化和吸附等诸多领域。基于TPOPs的固相微萃取(SPME)技术近年来引起了人们的极大兴趣,成为样品前处理技术领域的研究热点之一。该文简要地综述了近年来TPOPs的合成方法及其在固相微萃取领域的应用与发展,并对该领域研究进行了展望。

## Abstract (en)

The large surface area, adjustable pore structure, good thermal and chemical stabilities, and abundant *π*-electron systems make triazine-based porous organic polymers (TPOPs) as promising porous materials for gas storage, catalysis, energy conversion and adsorption. Recently, TPOPs have aroused ever-increasing interest and are considered as one of the research highlights in solid phase microextraction (SPME) and other sample pretreatment techniques. This minireview summarizes the recent advancements in the synthesis of TPOPs and their applications in SPME. The application prospects of the TPOPs in SPME and other sample pretreatment techniques are also presented.

三嗪基多孔有机材料(triazine-based porous organic polymers, TPOPs)是一类应用前景广阔的新型多孔材料。得益于其结构上1,3,5-三嗪环与芳环单体的共轭作用,三嗪基多孔有机材料骨架的能量降低,结构稳定性提高。目前,TPOPs以其多样的种类和性质、较大的比表面积、丰富的孔隙结构、良好的热和化学稳定性等特点而被广泛研究应用于诸多领域。TPOPs特殊的结构和优异的性能也在分析化学中显示出了良好的应用潜力,尤其是在固相微萃取(SPME)等样品前处理领域中^[1,2,3]^。本文对三嗪基多孔有机材料的合成以及它们在固相微萃取中的应用与发展进行了简要评述和展望。

## 1 三嗪基多孔有机材料的合成

目前,合成三嗪基多孔有机材料的方法主要有离子热法、超酸催化法和溶剂热法等。2008年,Kuhn等^[4]^首次以熔融的ZnCl_2_为溶剂和催化剂,以1,4-二氰基苯为单体,在离子热条件下三聚反应40 h,得到了三嗪基骨架材料(CTF)。随后,该课题组^[5]^在离子热条件下以2,6-二腈基萘为单体自聚合生成了CTF-2。鉴于传统的离子热法存在着反应温度高、反应时间长等问题,Zhang等^[6]^提出了微波辅助离子热法,他们以1,4-二氰基苯等为单体,在微波加热模式下进行的聚合反应可以在几十分钟内完成,所合成的CTFs具有较高的比表面积和孔隙率。该方法有效降低了反应温度,缩短了反应时间。Algara-Siller等^[7]^以双氰胺为单体采用离子热界面反应,合成了基于三嗪的石墨化碳氮化物(TGCN)的大尺寸结晶薄膜。Zhang等^[8]^通过NaCl/KCl熔盐的离子热法合成了具有三嗪七嗪共聚物的聚碳氮化物。根据共晶熔盐NaCl/KCl熔点的不同,可以调节聚合过程和优化聚合物结构。Wang等^[9]^以四氟对苯二甲腈为单体,在400 ℃熔融ZnCl_2_的电离热辅助下合成了全氟共价三嗪骨架化合物(FN-CTF-400),该材料表现出对电催化还原CO_2_的高选择性。

离子热法仍存在无机残留物过多等问题,2012年,Ren等^[10]^以超酸三氟甲磺酸(CF_3_SO_3_H)为催化剂,在室温和微波辅助条件下合成CTF。与离子热合成获得的黑色材料不同,三氟甲磺酸催化缩合后得到的材料是自由流动的荧光粉末,其吸收光谱和光致发光光谱取决于单体的选择。CF_3_SO_3_H催化三嗪聚合的反应条件较为温和,提高了反应条件的伸缩性。Zhu等^[11]^通过CF_3_SO_3_H超酸催化联苯二腈,在低温环境下(<100 ℃)合成了具有荧光特性的多孔三嗪骨架材料。Liu等^[12]^巧妙地在CH_2_Cl_2_/CF_3_SO_3_H溶剂界面上,以1,4-二氰基苯为单体合成了单层/少层的三嗪基多孔有机材料。随后,Liu等^[13]^以1,4-二氰基苯为单体,在CH_2_Cl_2_/CF_3_SO_3_H溶剂体系中通过一锅三氟乙酸催化三聚反应合成了结晶型CTF。经过液体超声后得到的CTFs用于钠离子电池表现出了良好性能,可实现CTFs的规模化生产。

考虑到早期离子热方法存在反应条件苛刻、单体类型有限等问题,基于多种缩合反应的溶剂热法逐渐发展成为合成TPOPs的新策略。Schwab等^[14]^在无催化剂的条件下通过三聚氰胺和醛类的席夫碱缩聚反应,成功地合成了一种高比表面积的席夫碱交联材料(Schiff base network, SNW)。此方法反应单体廉价,合成方法简单,反应条件温和。2016年,Gomes等^[15]^以1,3,5-三(4-氨基苯基)三嗪和1,3,5-三甲酰基间苯三酚为单体,通过席夫碱缩合反应合成了高结晶、高比表面积的共价有机骨架材料(covalent organic frameworks, COFs)。该材料有着良好的发光强度,并成功应用于硝基芳烃的化学传感。2017年,谭必恩等^[16]^以盐酸脒和不同的芳香醛为单体,采用席夫碱反应和Michael加成反应合成了多种CTFs。此方法避免了高温或强酸的反应,也不需要惰性气体保护,可大规模生产。另外,傅克(Friedel-Craft)反应以其条件温和、原料廉价、易于合成等特点,成为TPOPs的合成路线之一。Lim等^[17]^以AlCl_3_为催化剂,通过三聚氰氯与芳香族化合物的傅克反应制备了一系列TPOPs。这些材料的比表面积高达1266 m^2^/g,在常压和常温下表现出较高的CO_2_吸收率。Dey等^[18]^通过AlCl_3_催化三聚氯氰(CC)与三蝶烯或芴的傅克反应制备了两类CTFs,即CTF-TPC和CTF-FL。其中,CTF-TPC的比表面积高达1668 m^2^/g。Geng等^[19]^以甲烷磺酸为催化剂,通过2,4,6-三氯-1,3,5-三嗪与四苯基联苯胺的傅克反应合成了新型的富氮荧光共轭微孔聚合物TTPB。Troschke等^[20]^在AlCl_3_催化傅克反应体系中,选择三聚氯氰为三嗪节点,苯、萘、咔唑、1,3,5-三苯基苯和四苯基甲烷等富电子芳香族化合物为亲核试剂,通过机械研磨法快速合成了一系列CTFs。该方法为TPOPs的合成提供了一种无溶剂、省时和可扩展的替代方案。除席夫碱反应和傅克反应外,偶联反应以其反应条件温和、操作方便、易于合成等特点成为TPOPs合成的又一途径。2017年,Wang等^[21]^以Pd(PPh_3_)_2_Cl_2_为催化剂,将三聚氰尿氯(2,4,6-三氯三嗪)与芳基或乙烯基硼酸或二芳基硼酸在温和条件下进行高选择性顺序Suzuki偶联反应,合成了不对称芳基TPOPs。Guo等^[22]^以四苯基卟啉和三聚氰酸为单体,通过一锅Scholl偶联反应和傅克反应合成了卟啉基三嗪聚合物。制得的聚合物具有较大的CO_2_吸收容量(173 mg/g)和良好的CO_2_/N_2_选择性。Gu等^[23]^以三聚氰氯与三蝶烯、螺二芴为单体通过调节两个共存化学反应(即傅克反应和Scholl聚合反应)的竞争来调节聚合物的孔隙率和气体吸附性能。虽然上述傅克反应和偶联反应的合成条件较离子热法简单、温和,但是所合成的TPOPs结晶度依然较低。

为了提高TPOPs的晶体性,谭必恩等^[24]^以二甲基亚砜为溶剂,进行缩聚反应,通过控制醇类单体缓慢氧化成醛,以达到控制反应速率的目的,从而获得高结晶度的CTFs。结晶性CTFs的结晶度、热稳定性和层状结构均有较大改善。相对低晶态和非晶态CTFs,晶态CTFs表现出更高的光催化性能。该课题组^[25]^随后通过直接控制醛类单体的加料速率来合成高结晶度的CTFs。该晶态CTFs对NO的脱除率优于无定形CTFs材料,甚至远优于石墨相氮化碳(g-C_3_N_4_)。与离子热法、超强酸催化法、傅克反应等典型合成方法相比,上述控制加料速率法具有结晶度可控、生产方便、反应条件温和等优点。这种开放式系统的进料速度控制方法有可能用于TPOPs的大规模生产。2020年,谭必恩等^[26]^在强碱的辅助下,通过含苄氨基单体的缩聚反应合成了结晶型CTFs。此CTFs材料具有较高的结晶度和优异的亲水性能,光催化析氢的最高析氢速率比其他方法都高。

## 2 三嗪基多孔有机材料在固相微萃取中的应用

Guo等^[27]^以2,4,6-三苯氧基-1,3,5-三嗪为单体,制备了含有微孔和中孔的双孔COFs。该材料用于固相微萃取测定邻苯二甲酸酯类塑化剂,展现了良好的应用前景。贾琼等^[28]^以三聚氰胺和4,4-联苯二硼酸作为单体制备了多孔芳香族骨架材料(porous aromatic framework, PAF),采用物理黏合法制备了基于PAF的固相微萃取纤维,建立了化妆品中防腐剂和抗氧化剂的固相微萃取测定方法。该材料对分析物良好的吸附效率归因于PAF较高的疏水性、*π*-*π*堆积相互作用,以及PAF的硼羟基与分析物之间形成的氢键作用。本课题组^[29]^通过三聚氯氰与哌嗪的一步亲核取代反应合成了多孔芳香族骨架材料PAF-6,并将基于PAF-6涂层的固相微萃取方法成功用于测定水样中的多环芳烃。实验结果表明,*π*-*π*堆积和疏水相互作用对萃取均有影响,但前者占主导地位。Hou等^[30]^设计了一种新型氟功能化的三嗪基COF-F-1,采用物理黏合法制成了固相微萃取探针(COFs-SPME)。将COFs-SPME和纳米电喷雾电离质谱(nanoESI-MS)相结合,建立了环境和生物样品中痕量全氟烷基物质和多氟烷基物质的超灵敏快速分析方法。2019年,本课题组^[31]^通过三聚氯氰和对三联苯的偶联反应制备了一种具有大比表面积、高溶剂稳定性和良好热稳定性的PAF-56P,通过物理黏合法制备了PAF-56P涂层SPME纤维,借助气相色谱-质谱检测建立了高效富集水果和蔬菜样品中的三唑类杀菌剂。Li等^[32]^通过三聚氰胺与对苯二甲醛的席夫碱反应,在二氧化硅纳米球表面原位制备了TPOPs材料,并将该复合材料纳米球物理黏合在不锈钢丝上制成固相微萃取纤维。随后将此纤维置于聚醚醚酮管中进行管内固相微萃取多环芳烃,取得了较好的萃取效果。

除了上述物理黏合法制备固相微萃取纤维之外,李攻科等^[33]^采用多层交联化学键合方法,通过SNW-1骨架内的-NH_2_和石英纤维上烷氧试剂功能化的环氧基共价键合制备出SNW-1固相微萃取纤维,并成功地用于茶叶和烟丝样品中的多环芳烃和挥发性脂肪酸的分析。SNW-1与环氧基形成的共价键和桥接显著提高了涂层的稳定性和均匀性,使得该固相微萃取纤维表现出较强的化学稳定性。本课题组^[34]^将水热法合成的SNW-1化学键合到不锈钢丝上,制备了SNW-1涂层固相微萃取纤维。基于SNW-1的固相微萃取方法在萃取蜂蜜中的氯酚时取得了较高的富集倍率和较低的检出限。得益于SNW-1自身的*π*键体系和介孔特征(2.80 nm),与直链烷烃相比,SNW-1展现了对氯酚和多环芳烃良好的吸附选择性。

考虑到上述研究主要集中在合成新型的三嗪基多孔有机材料和改变固相微萃取纤维的涂层方法等内容上,缺乏对于TPOPs自身孔径结构、*π*-电子体系等对特定分析物萃取效率影响等深入分析。我们通过调整三聚氯氰和三苯胺(TPA)的单体浓度,以甲磺酸催化的傅克反应,制备了3种具有不同孔隙特性的TPOPs(CC-TPA-1, CC-TPA-2和CC-TPA-3)^[35]^。实验结果表明,3种TPOPs对分析物有机氯农药分子的吸附能力不同,其中具有最大比表面积和孔体积的CC-TPA-1的萃取效率最高。另外,我们以三聚氯氰和联苯(BP)、对三联苯(TP)和对四联苯(QP)为单体,通过傅克反应合成3个结构相似、但*π*电子体系不同的CTFs CC-BP、CC-TP和CC-QP^[36]^。与CC-BP和CC-TP相比,具有较大表面积和*π*电子体系的CC-QP对多环芳烃的固相微萃取效率最高。

此外,TPOPs衍生碳材料由于较高的比表面积、良好的化学和热稳定性逐渐受到关注。Ben等^[37]^制备了一系列PAF-1衍生碳材料,这些材料表现出显著的CO_2_吸收容量和极高的CO_2_/H_2_、CO_2_/N_2_和CO_2_/CH_4_吸附选择性。本课题组^[38]^以PAF-6为模板和碳源,KOH为化学活化剂,制备了多孔碳材料。将基于PAF-6衍生多孔碳材料的固相微萃取用于测定水和土壤样品中直链烷烃,获得较宽的线性范围、较低的检出限和良好的重复性。表1列出了近年来TPOPs及其衍生碳材料在固相微萃取中的应用。

**表1:** 三嗪基多孔有机材料在固相微萃取中的应用

Coating	Samples	Analytes	Analytical method	LOD	Linear range	Ref.
TPT-COF	juice	PAEs	GC-FID	5-95 ng/L	0.01-100 μg/L	[27]
PAF	cosmetics	antioxidants	GC-FID	0.12-0.22 μg/L	0.02-500 mg/L	[28]
		preservatives		0.19-0.30 μg/L		
PAF-6	water	PAHs	GC-MS	0.8-4.2 ng/L	1.6-4000 ng/L	[29]
COF-F-1	water	PFASs	nanoESI-MS	0.02-0.8 ng/L	1-5000 ng/L	[30]
PAF-56P	fruits and vegetables	triazole fungicides	GC-MS	0.15-1.70 ng/g	2.50-250 ng/g	[31]
TOPs@SiO_2_	water	PAHs	HPLC	0.003-0.010 μg/L	0.010-20 μg/L	[32]
SNW-1	tea leaf, tobacco	VFAs	GC-MS	0.014-0.026 μg/L	0.05-10 μg/L	[33]
	honey	phenols	GC-MS	0.06-0.2 ng/g	0.1-100 ng/g	[34]
CC-TPA	apple, peach and pear	organochlorine pesticides	GC-ECD	0.032-0.090 ng/g	0.11-20 ng/g	[35]
CC-QP	honey	PAHs	GC-MS	0.03-0.19 ng/g	0.10-100 ng/g	[36]
PAF-6-NPC	water	n-alkanes	GC-FID	0.8-6 ng/L	5-2000 ng/L	[38]
	soil			0.01-0.05 ng/g	0.05-20 ng/g	

TPT-COF: 2,4,6-triphenoxy-1,3,5-triazine-covalent organic framework; PAF: porous aromatic framework; COF-F-1: fluorine-functionalized COF; TOPs@SiO_2_: triazine-based organic polymers@SiO_2_; SNW-1: Schiff base network-1; CC-TPA: cyanuric chloride-triphenylamine; CC-QP: cyanuric chloride-*p*-quaterphenyl; PAF-6-NPC: PAF-6 derived nanoporous carbon; PAEs: phthalic acid esters; PAHs: polycyclic aromatic hydrocarbons; PFASs: per- and polyfluoroalkyl substances; VFAs: volatile fatty acids; ECD: electron capture detection.

## 3 总结与展望

本文简要综述了三嗪基多孔有机材料的合成方法及其在固相微萃取领域的应用与发展。从早期的ZnCl_2_离子热法、超酸催化法,到席夫碱反应、傅克反应和偶联反应等溶剂热法,再到后来为提高TPOPs结晶性涌现出的新方法,三嗪基多孔有机材料的合成方法不断地革新进步。上述基于三嗪基多孔有机材料的固相微萃取研究初步显示TPOPs在样品预处理领域具有良好的应用前景。然而,相较于种类繁多、数目庞大的三嗪基多孔有机材料而言,当前仅仅有少数TPOPs用于固相微萃取等样品预处理技术中。结合其特殊的结构和优异的性能,TPOPs用于固相微萃取等样品预处理技术中仍有很大的拓展空间,今后在以下几个方面仍需进一步加强研究:①就三嗪基多孔有机材料的合成而言,现有的离子热法存在条件苛刻、费时、单体类型有限、无机残留物过多等问题。而溶剂热法虽然条件温和,操作简易,但是所合成的TPOPs结晶度有待提高。因此,开发绿色、简便的TPOPs合成方法,合成晶体性良好、稳定性高的COFs将会有效促进三嗪基多孔有机材料在固相微萃取等样品预处理领域的应用进展。②鉴于部分TPOPs的稳定性较差而大大限制了该材料在样品预处理领域的应用,近年来出现了由三嗪基多孔有机材料与其他功能材料结合而形成的复合材料,不仅提高了TPOPs的稳定性,还扩增了TPOPs的种类和性质,同时弥补了其中单一相的功能应用受限的问题,因此开展基于TPOPs复合材料样品预处理的研究将是一个很重要并且前景广阔的方向。③三嗪基多孔有机材料的孔径结构、*π*-电子体系和特殊官能团等对特定分析物选择性吸附方面的研究亟待加强,结合密度泛函理论(density functional theory, DFT)模拟计算,构建预组装过程的理论模型,揭示TPOPs选择性吸附机理也将是这一领域的重要研究方向。综上所述,三嗪基多孔有机材料具有比表面积较大、孔径可调、稳定性较高、结构容易修饰等优点,有望解决一些传统吸附剂材料难以解决的分离富集问题。三嗪基多孔有机材料的设计与开发是一项具有重要学术价值和应用前景的研究工作,该材料将在固相微萃取等样品预处理技术中不断发展进步。
